# Supplementary material for: Effect of Lacticaseibacillus paracasei Strain Shirota on Improvement in Depressive Symptoms, and Its Association with Abundance of Actinobacteria in Gut Microbiota
Source: Microorganisms. 2021 May 10;9(5):1026. doi: 10.3390/microorganisms9051026 (PMC8150707; doi:10.3390/microorganisms9051026)
Supplement: Supplementary file 1 [file microorganisms-09-01026-s001.zip › microorganisms-1189554-supplementary.pdf]

Table S1. Changes in the gut microbiota.

Bacterial target [former name (sg, subgroup)]		Bacterial count (log_{10} cells/g of stool)[a]			*p*-value[d]
		0W	6W	12W	
Total bacteria[b]		10.6 (10.4, 10.8)	10.7 (10.5, 10.9)	10.6 (10.4, 10.9)	0.086
Clostridium coccoides group		9.8 (9.3, 10.1)	9.8 (9.6, 10.1)	9.9 (9.5, 10.2)	0.904
Clostridium leptum subgroup		9.7 (9.3, 9.9)	9.7 (9.4, 9.9)	9.6 (9.1, 9.9)	0.148
Bacteroides fragilis group		9.6 (9.1, 10.1)	9.9 (9.4, 10.2)	9.9 (9.4, 10.2)	0.235
Bifidobacterium		9.9 (8.7, 10.4)	10.1 (9.7, 10.7)	9.8 (9.3, 10.5)	0.073
Atopobium cluster		9.7 (8.9, 9.9)	9.8 (8.8, 10.1)	9.6 (9.0, 9.9)	0.408
Prevotella		6.0 (2.5, 8.0)	2.5 (2.5, 6.8)	2.5 (2.5, 7.0)	1.000
Clostridium difficile		1.5 (1.5, 1.5)	1.5 (1.5, 1.5)	1.5 (1.5, 1.5)	NA
Clostridium perfringens		1.4 (1.4, 1.4)	1.4 (1.4, 1.4)	1.4 (1.4, 1.4)	0.368
Total lactobacilli[c]		6.4 (5.5, 7.3)	7.9 (7.7, 8.1)	7.8 (7.5, 7.9)	< 0.001**
Enterobacteriaceae		6.4 (5.6, 6.8)	6.5 (5.7, 7.0)	6.4 (5.5, 7.3)	0.881
Enterococcus		5.3 (2.6, 6.7)	5.2 (4.4, 6.3)	5.1 (4.3, 6.3)	0.984
Staphylococcus		4.9 (4.3, 5.1)	4.8 (4.3, 5.0)	5.0 (4.6, 5.4)	0.785
<u>Subpopulation of lactobacilli</u>					
Lacticaseibacillus	[*Lactobacillus casei* sg]	4.1 (2.0, 5.6)	7.8 (7.7, 8.0)	7.6 (7.5, 7.9)	< 0.001**
Lactobacillus	[*Lactobacillus gasseri* sg]	5.7 (5.0, 6.4)	4.9 (2.2, 5.7)	5.0 (1.7, 6.0)	0.219
Lactiplantibacillus	[*Lactobacillus plantarum* sg]	1.5 (1.5, 3.5)	1.5 (1.5, 3.5)	1.5 (1.5, 3.6)	0.975
Limosilactobacillus except *L. fermentum*	[*Lactobacillus reuteri* sg]	4.8 (2.3, 5.6)	4.0 (1.8, 4.8)	3.9 (1.8, 4.7)	0.144
Limosilactobacillus fermentum	[*Lactobacillus fermentum*]	2.5 (2.5, 2.5)	2.5 (2.5, 2.5)	2.5 (2.5, 2.5)	0.291
Liquorilactobacillus and *Ligilactobacillus*	[*Lactobacillus ruminis* sg]	1.5 (1.5, 4.9)	1.5 (1.5, 4.5)	1.5 (1.5, 4.5)	0.216
Latilactobacillus	[*Lactobacillus sakei* sg]	4.3 (2.2, 5.0)	4.5 (3.7, 4.8)	4.3 (3.3, 4.7)	0.834
Levilactobacillus brevis	[*Lactobacillus brevis*]	2.0 (2.0, 2.0)	2.0 (2.0, 2.0)	2.0 (2.0, 2.0)	0.368

[a] The count (log_{10} cells/g of stool) of each bacterial target was measured by the RT-qPCR assay targeting rRNA molecules, and data are expressed as median (lower quartile, upper quartile) of the count. The counts of "not detected" samples were regarded as representing half the detection limit in the statistical analysis. [b] The total bacterial count obtained by RT-qPCR is expressed as the sum of the counts of 10 groups and 2 species. [c] The count of total lactobacilli is expressed as the sum of the counts of the 8 subpopulations. [d] The Friedman test was used for the multiple comparison between the time points (**, $p < 0.010$; NA, not applicable).

Table S2. Comparison of the demographic and clinical characteristics between the responder subgroups, based on the percent change in the HAM-D21 total score.

		Responder subgroup[a]		*p*-value[b]
		Responder	Non-responder	
Sex	Female, *n* (%)	5 (62.5)	9 (90.0)	0.275
	Male, *n* (%)	3 (37.5)	1 (10.0)	
Disease	MDD, *n* (%)	7 (87.5)	8 (80.0)	1.000
	BD, *n* (%)	1 (12.5)	2 (20.0)	
Age, years	Mean ± SD	41.9 ± 12.0	39.6 ± 11.4	0.778
	95% CI	[31.8, 51.9]	[31.4, 47.8]	
	Median (min, max)	47 (23, 55)	38 (21, 54)	
Education, years	Mean ± SD	13.7 ± 2.3	16.1 ± 1.4	0.015*
	95% CI	[11.8, 15.6]	[15.1, 17.1]	
	Median (min, max)	14 (9, 16)	16 (14, 18)	
BMI, kg/m^2	Mean ± SD	22.32 ± 2.66	21.38 ± 5.27	0.305
	95% CI	[20.10, 24.54]	[17.61, 25.14]	
	Median (min, max)	23.0 (18.3, 25.3)	20.2 (16.5, 34.8)	
HAM-D21 total score	Mean ± SD	16.8 ± 4.1	18.4 ± 4.3	0.303
	95% CI	[13.4, 20.1]	[15.3, 21.5]	
	Median (min, max)	17 (11, 22)	19 (12, 24)	
BDI score	Mean ± SD	27.5 ± 12.9	34.7 ± 8.6	0.210
	95% CI	[16.7, 38.3]	[28.5, 40.9]	
	Median (min, max)	29 (0, 44)	36 (18, 45)	
IBS diagnosis	Normal, *n* (%)	2 (25.0)	3 (30.0)	0.424
	FBD, *n* (%)	4 (50.0)	2 (20.0)	
	IBS, *n* (%)	2 (25.0)	5 (50.0)	
STAI trait, class[c]	High, *n* (%)	2 (25.0)	2 (20.0)	1.000
	Very high, *n* (%)	6 (75.0)	8 (80.0)	
Medication	Receiving, *n* (%)	6 (75.0)	9 (90.0)	0.559
	Non-receiving, *n* (%)	2 (25.0)	1 (10.0)	

[a] The responder subgroups were assigned based on the percent change in the HAM-D21 total score over the 12-week intervention period. The patients whose percent change was -50.0% or less were designated as "responder" ($n = 8$), with the remaining participants designated as "non-responder" ($n = 10$) [b] Wilcoxon's rank sum test, and Fisher's exact test for 2 × 3 or 2 × 2 contingency tables, were used for the inter-group comparison of ratio and nominal data, respectively (*, $p < 0.050$). [c] No subjects scored "very low", "low", or "normal".

Table S3. Characteristics of the gut microbiota in the responder subgroups, based on the percent change in the HAM-D21 total score.

**Bacterial target**[a]	**Responder (n = 8)**[b]			**Non-responder (n = 10)**[b]		
	**0W**	**6W**	**12W**	**0W**	**6W**	**12W**
Total bacteria	10.8 (10.6, 11.0)	10.8 (10.6, 11.0)	10.7 (10.5, 11.0)	10.5 (10.4, 10.6)	10.6 (10.5, 10.8)	10.5 (10.3, 10.8)
Clostridium coccoides group	9.7 (9.5, 10.1)	9.9 (9.6, 10.0)	9.9 (9.6, 10.2)	9.9 (9.4, 10.1)	9.8 (9.6, 10.2)	9.9 (9.5, 10.1)
Clostridium leptum subgroup	9.5 (9.3, 9.8)	9.5 (9.3, 9.8)	9.3 (9.0, 9.9)	9.8 (9.4, 9.9)	9.8 (9.7, 10.0)	9.7 (9.3, 10.0)
Bacteroides fragilis group	9.5 (9.0, 9.7)	9.9 (9.6, 10.0)	10.0 (9.3, 10.2)	10.0 (9.4, 10.2)	10.1 (9.4, 10.2)	9.9 (9.7, 10.2)
Bifidobacterium	10.4 (10.1, 10.6) *	10.3 (9.9, 10.7)	10.0 (9.8, 10.8)	9.5 (7.6, 9.9) *	9.9 (9.4, 10.5)	9.5 (9.2, 10.1) †
Atopobium cluster	9.9 (9.8, 10.0) *	10.1 (9.5, 10.2)	9.7 (9.1, 10.0)	9.2 (8.6, 9.6) *	9.5 (8.8, 10.0)	9.4 (8.6, 9.8)
Prevotella	6.2 (2.5, 8.4)	2.5 (2.5, 7.3)	2.5 (2.5, 7.5)	5.7 (2.5, 6.6)	4.6 (2.5, 6.8)	2.5 (2.5, 7.0)
Clostridium difficile	1.5 (1.5, 1.5)	1.5 (1.5, 1.5)	1.5 (1.5, 1.5)	1.5 (1.5, 1.5)	1.5 (1.5, 1.5)	1.5 (1.5, 1.5)
Clostridium perfringens	1.4 (1.4, 1.4)	1.4 (1.4, 1.4)	1.4 (1.4, 1.4)	1.4 (1.4, 1.4)	1.4 (1.4, 1.4)	1.4 (1.4, 1.4)
Total lactobacilli	6.8 (5.8, 7.4)	7.9 (7.8, 8.1)	7.8 (7.6, 7.9) †	6.0 (5.5, 6.8)	7.8 (7.6, 8.0)	7.7 (7.5, 7.9) †
Enterobacteriaceae	6.5 (5.3, 6.9)	6.4 (5.3, 7.0)	6.7 (5.7, 7.2)	6.3 (5.7, 6.4)	6.6 (5.7, 6.9)	6.2 (5.5, 7.1)
Enterococcus	6.0 (5.0, 6.8)	5.3 (4.4, 5.8)	5.1 (4.6, 6.9)	4.7 (2.0, 5.5)	4.7 (4.4, 6.3)	5.0 (2.6, 6.0)
Staphylococcus	4.5 (3.7, 5.1)	5.0 (4.4, 5.1)	5.1 (5.0, 5.4)	4.9 (4.6, 5.0)	4.6 (4.3, 5.0)	4.7 (4.4, 5.3)

[a] The count ($\log_{10}$ cells/g of stool) of each bacterial target was measured by the RT-qPCR assay targeting rRNA molecules, and data are expressed as median (lower quartile, upper quartile) of the count. The counts of "not detected" samples were regarded as representing half the detection limit in the statistical analysis. [b] Wilcoxon's rank sum test was used for the inter-group comparison between "responder" and "non-responder" at each time point (*, $p < 0.050$). The Friedman test was used for the multiple comparison between the time points in each subgroup (†, $p < 0.050$).
